# Supplementary figures and images for: Robotic search for optimal cell culture in regenerative medicine
Source: eLife. 2022 Jun 28;11:e77007. doi: 10.7554/eLife.77007 (PMC9239686; doi:10.7554/eLife.77007)

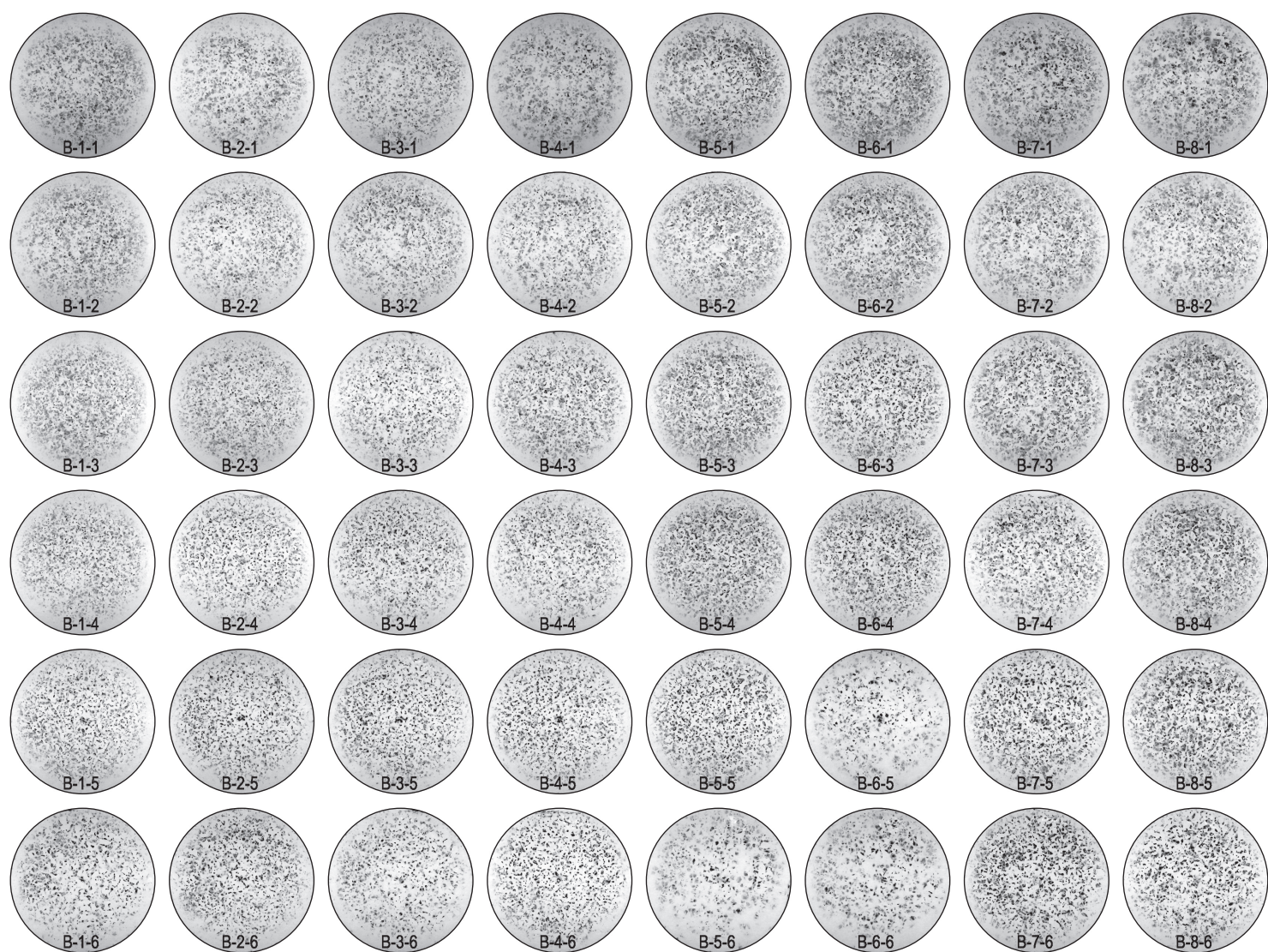

Supplement: Figure 2—source data 1. — Images acquired on Day 34 of the baseline experiment; images of the bottom of the well with cultured cells, cropped to the size of the well. These 8-bit images were adjusted to a minimum and maximum contrast value of 100 and 150, respectively. IDs on the bottom indicate 'B (baseline) - Plate No. - Well No.'. [file elife-77007-fig2-data1.zip › Figure_2_source_data_1.pdf]

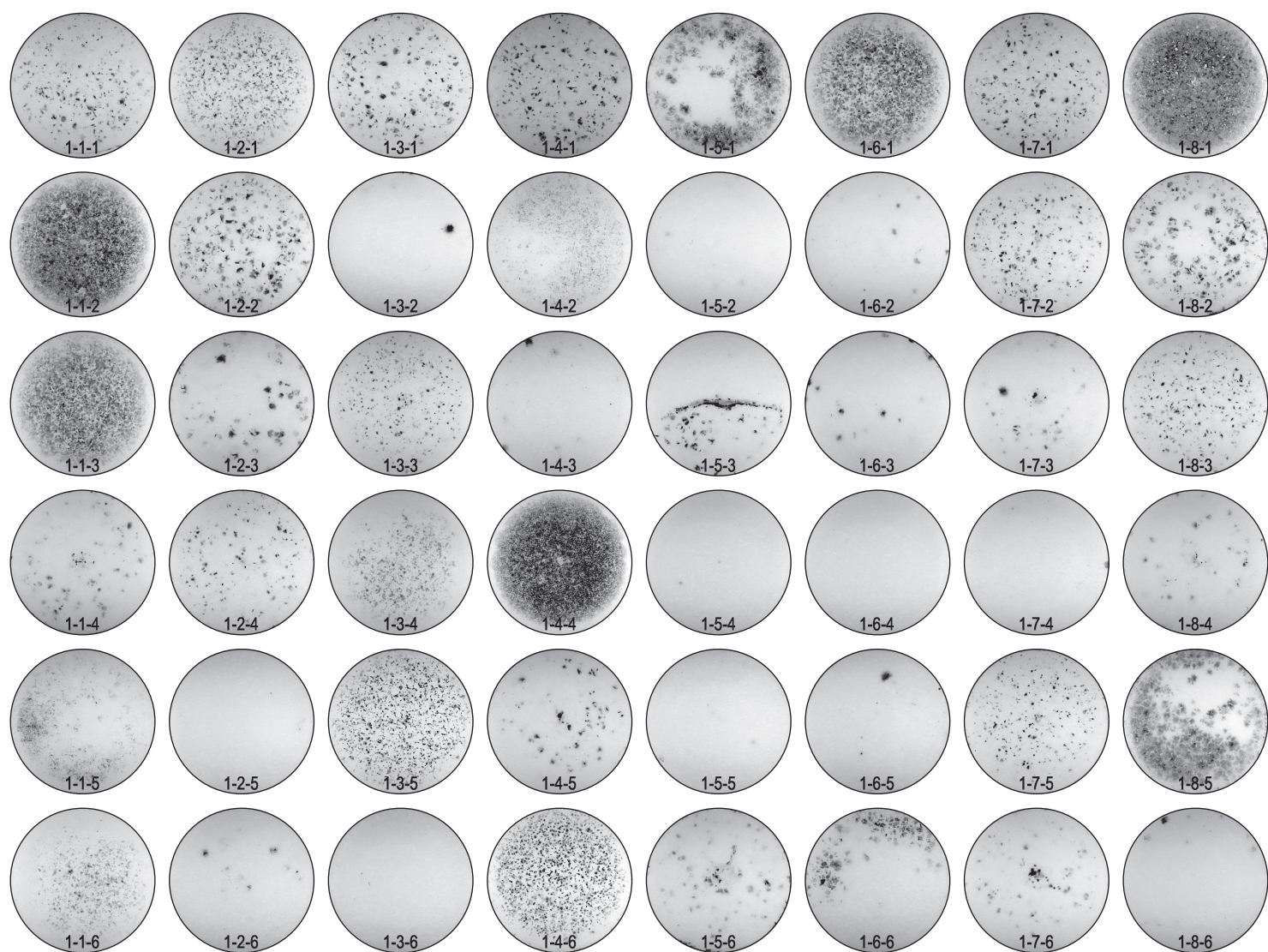

Supplement: Figure 4—source data 1. — Images acquired on Day 34 of the round 1 experiment; images of the bottom of the well with cultured cells, cropped to the size of the well. These 8-bit images were adjusted to a minimum and maximum contrast value of 100 and 150, respectively. ID labeling on the bottom indicates '1 (round 1) - Plate No. - Well No.'. [file elife-77007-fig4-data1.zip › Figure_4_source_data_1.pdf]

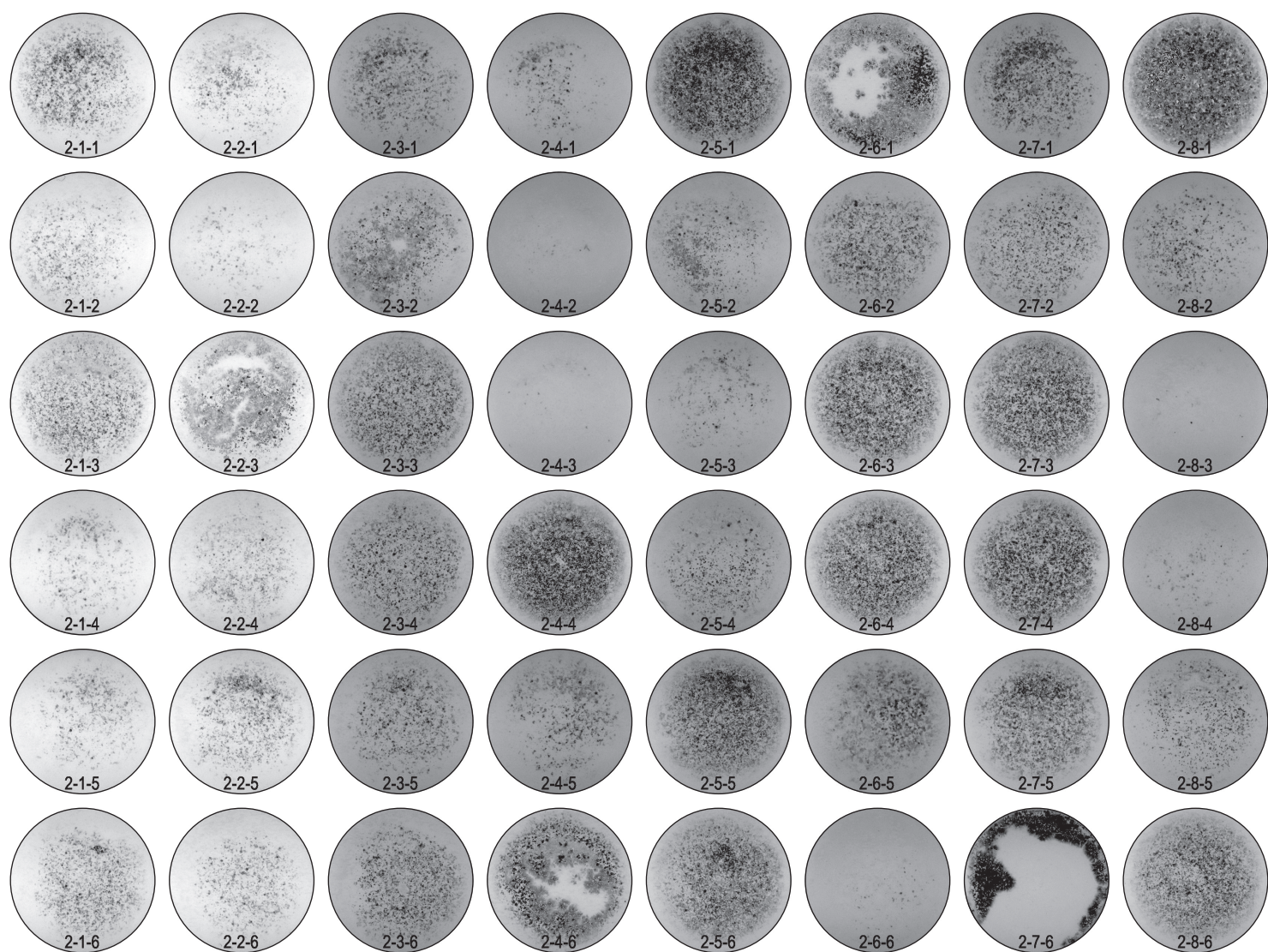

Supplement: Figure 4—source data 2. — Images acquired on Day 34 of the round 2 experiment; images of the bottom of the well with cultured cells, cropped to the size of the well. These 8-bit images were adjusted to a minimum and maximum contrast value of 100 and 150, respectively. ID labeling on the bottom indicates '2 (round 2) - Plate No. - Well No.'. [file elife-77007-fig4-data2.zip › Figure_4_source_data_2.pdf]

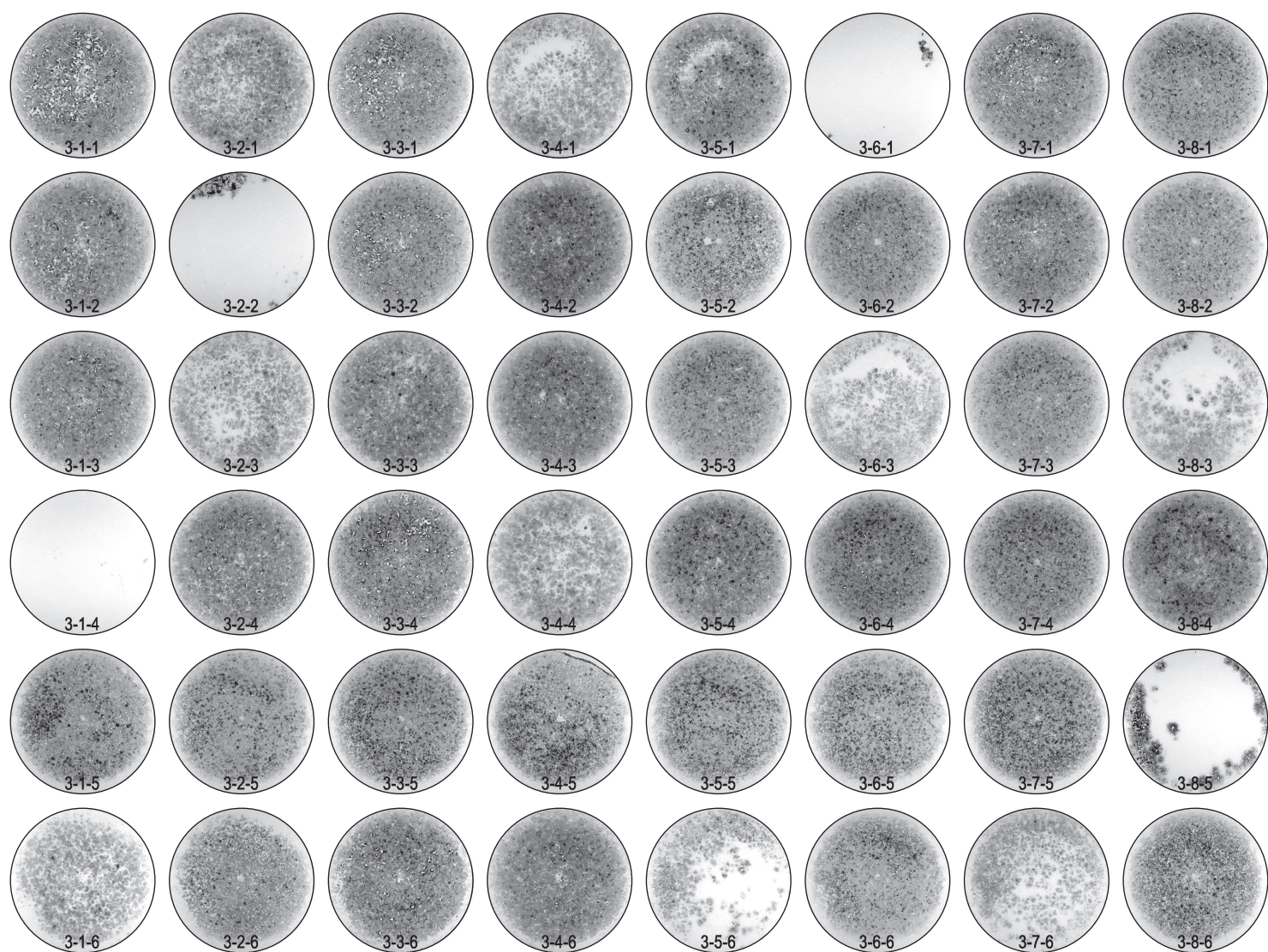

Supplement: Figure 4—source data 3. — Images acquired on Day 34 of the round 3 experiment: images of the bottom of the well with cultured cells, cropped to the size of the well. These 8-bit images were adjusted to a minimum and maximum contrast value of 100 and 150, respectively. ID labeling on the bottom indicates '3 (round 3) - Plate No. - Well No.'. [file elife-77007-fig4-data3.zip › Figure_4_source_data_3.pdf]

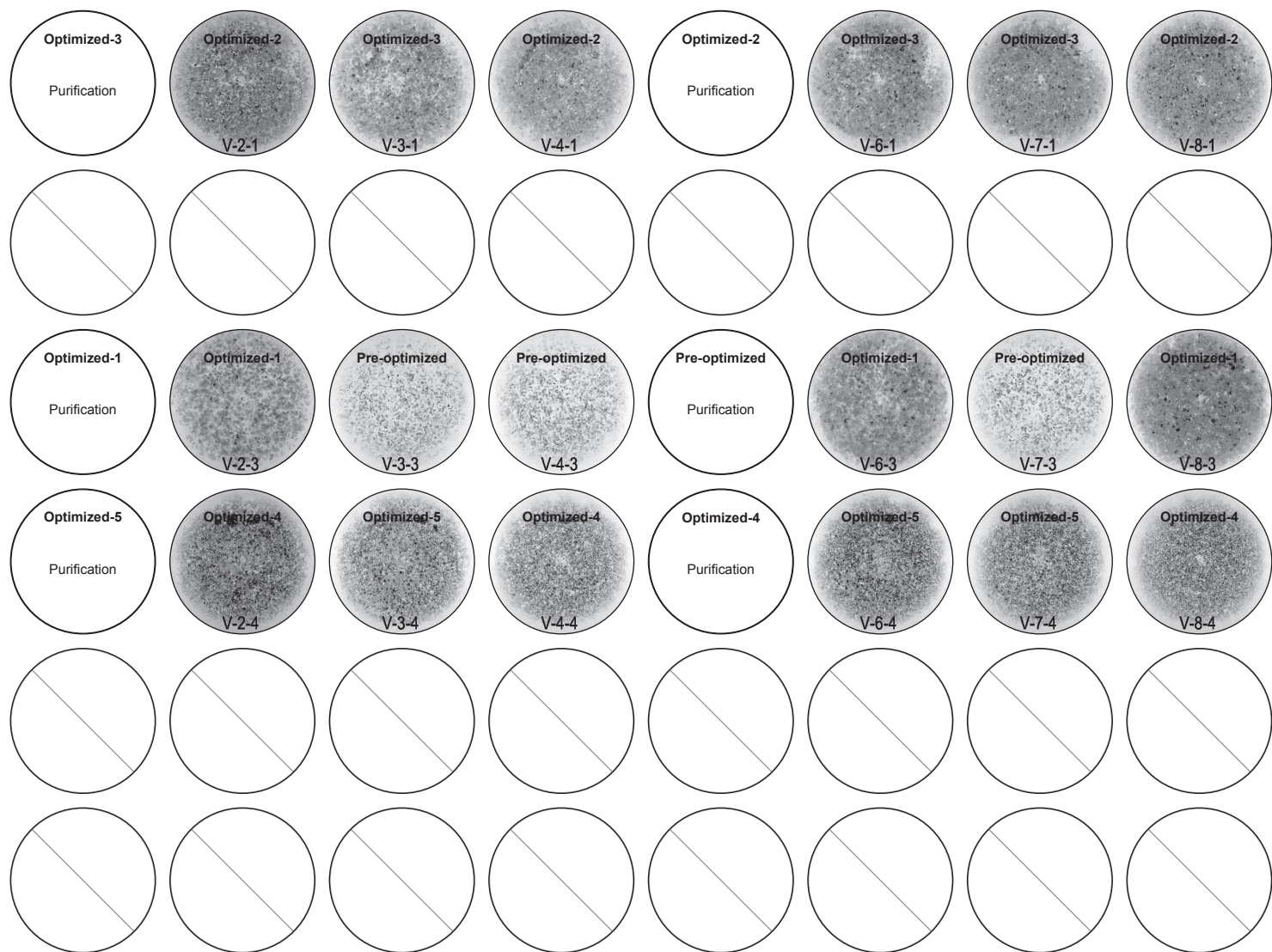

Supplement: Figure 5—source data 1. — Images acquired on Day 34 of the validation experiment: images of the bottom of the well with cultured cells, cropped to the size of the well. These 8-bit images were adjusted to a minimum and maximum contrast value of 80 and 125, respectively. Sample names on the top correspond to Figure 5A. ID labeling on the bottom indicates 'V (validation) - Plate No. - Well No.'. Wells 2, 5, and 6 were not subjected to the validation experiments. Plates 1 and 5 were used for cell biological analysis and were not evaluated using images. [file elife-77007-fig5-data1.zip › Figure_5_source_data_1.pdf]
